# Quasispecies in population of compositional assemblies

**DOI:** 10.1186/s12862-014-0265-1

**Published:** 2014-12-30

**Authors:** Renan Gross, Itzhak Fouxon, Doron Lancet, Omer Markovitch

**Affiliations:** Department of Molecular Genetics, Weizmann Institute of Science, Rehovot, 76100 Israel; Interdisciplinary Computing and Complex Bio-Systems research group, School of Computing Science, Newcastle University, Newcastle upon Tyne, NE1 7RU UK

**Keywords:** Origin of life, Composomes, Lipid world, GARD, Quasispecies, Error threshold, Compositional information, Composition, Sequence

## Abstract

**Background:**

The quasispecies model refers to information carriers that undergo self-replication with errors. A quasispecies is a steady-state population of biopolymer sequence variants generated by mutations from a master sequence. A quasispecies error threshold is a minimal replication accuracy below which the population structure breaks down. Theory and experimentation of this model often refer to biopolymers, e.g. RNA molecules or viral genomes, while its prebiotic context is often associated with an RNA world scenario. Here, we study the possibility that compositional entities which code for compositional information, intrinsically different from biopolymers coding for sequential information, could show quasispecies dynamics.

**Results:**

We employed a chemistry-based model, graded autocatalysis replication domain (GARD), which simulates the network dynamics within compositional molecular assemblies. In GARD, a compotype represents a population of similar assemblies that constitute a quasi-stationary state in compositional space. A compotype's center-of-mass is found to be analogous to a master sequence for a sequential quasispecies. Using single-cycle GARD dynamics, we measured the quasispecies transition matrix (Q) for the probabilities of transition from one center-of-mass Euclidean distance to another. Similarly, the quasispecies’ growth rate vector (A) was obtained. This allowed computing a steady state distribution of distances to the center of mass, as derived from the quasispecies equation. In parallel, a steady state distribution was obtained via the GARD equation kinetics. Rewardingly, a significant correlation was observed between the distributions obtained by these two methods. This was only seen for distances to the compotype center-of-mass, and not to randomly selected compositions. A similar correspondence was found when comparing the quasispecies time dependent dynamics towards steady state. Further, changing the error rate by modifying basal assembly joining rate of GARD kinetics was found to display an error catastrophe, similar to the standard quasispecies model. Additional augmentation of compositional mutations leads to the complete disappearance of the master-like composition.

**Conclusions:**

Our results show that compositional assemblies, as simulated by the GARD formalism, portray significant attributes of quasispecies dynamics. This expands the applicability of the quasispecies model beyond sequence-based entities, and potentially enhances validity of GARD as a model for prebiotic evolution.

**Electronic supplementary material:**

The online version of this article (doi:10.1186/s12862-014-0265-1) contains supplementary material, which is available to authorized users.

## Background

### The quasispecies model

The quasispecies theory describes the replication of asexual replicators at high error rate, and was first proposed to describe error-prone replication of primitive information-carrying macromolecules at the origin of life [1,2]. A quasispecies is often viewed as a steady-state population of variant biopolymer sequences, generated by mutations from a sequence [2-4]. This replication with mutation can lead to a population with a different dominant sequence than the original one, even if the original had the highest replication rate, i.e. highest fitness. As such, the quasispecies model is an example of how selection and evolution can arise from simple kinetic underpinnings [4]. Selection acts on the population as a whole rather than on the individual members [5].

While the theory is general to replication, a widely used application of quasispecies is in describing RNA viruses, which have low replication fidelity with measured high mutations rates [6-10], though the model’s validity for some RNA viruses has been a topic of dispute [7,9,11-13]. Other biological applications of quasispecies are to the multiple laboratory instances of the Chinese hamster ovary cell line [14] and to catalytic RNA molecules [15,16].

Using the quasispecies equation [2], it is possible to quantify an error threshold which relates the amount of information a replicating system can store to its single digit error probability [4,8,17]. The error threshold is defined as the minimum accuracy of replication which is required in order to preserve the information of the selected state of the system, beyond which the population structure breaks down. When the genotype-phenotype map involves redundancy (i.e. more than one genotype give rise to the fittest phenotype), the error threshold can be formulated in terms of phenotypes, and it the population can sustain a lower degree of replication accuracy [18,19]. As RNA viruses replicate with relatively high mutations rates [10], they are susceptible to conditions which increase their mutation rates to push them beyond the error catastrophe [20-22], a process parallel to extinction by the direct induction of deleterious mutations [23,24]. The error catastrophe path not only supports the quasispecies nature of RNA viruses, but is also an example of a relation between modeling and experiments.

### Sequential versus compositional information

Biological systems have two types of information. The first is the well-established sequence-based information, as manifested in biopolymers such as DNA, RNA and proteins. The second information type is compositional information, which plays a parallel central role in biological systems [25-29,54]. Composition is formally defined as a vector *V* whose elements are the counts or concentrations of molecular types. In an example, the identity of a living cell can be defined, to an extent, by the counts of all its RNA types (transcriptome) and proteins types (proteome) [30-35]. Compositional information is intrinsically different from sequence-based information, and the total number of different possible compositions, for a given alphabet size of N_G_ and a total count of N_max_ molecules in *V* is: $$ \left(\begin{array}{l}{N}_G+{N}_{max}-1\\ {}\kern3.5em {N}_{max}\end{array}\right) $$, while the total number of different sequences of a string of length N_max_ is: N_G_^Nmax^.

There are significant differences between sequential and compositional entities. For one, biopolymer sequence information is digitally encodable but compositional information is not, which may be viewed as a key difference between chemistry and biology. In the realm of polymeric entities, a point mutation is the replacement of a monomer type in a particular sequence position, necessitating the breaking and formation of covalent bonds. For compositional entities, a point mutation is the “random access” exchange of a molecule of a given type with a molecule of another type. Further, for sequences, the probability of a mutation at a specific location only depends on sequence length and not on the specific sequence. In contrast, for compositions such probability depends both on the size and the actual composition of the entity. For a composition with N_G_ = 2 and N_max_ = m + n: A_m_B_n_, the probability of a mutational transition to A_m+1_B_n-1_ is m/(m + n). Finally, for sequences, replication entails the template-based synthesis of a polymeric strand. In clear difference, a compositional entity undergoes replication/reproduction via composition-preserving growth, facilitated by a network of “many-to-many” molecular interactions, followed by fission [36]. In some formal respect, this is true also for present-day living cells. For example, a crucial step prior to cell division is the biosynthetic doubling of the compositional counts of the proteins that characterize a given cell type. But such similarity cannot be taken very far, since present day cells divide by a highly complex and completely genetically controlled mechanism. Compositional entities have been invoked in models for early evolution [28,37-40].

**Figure 1:**
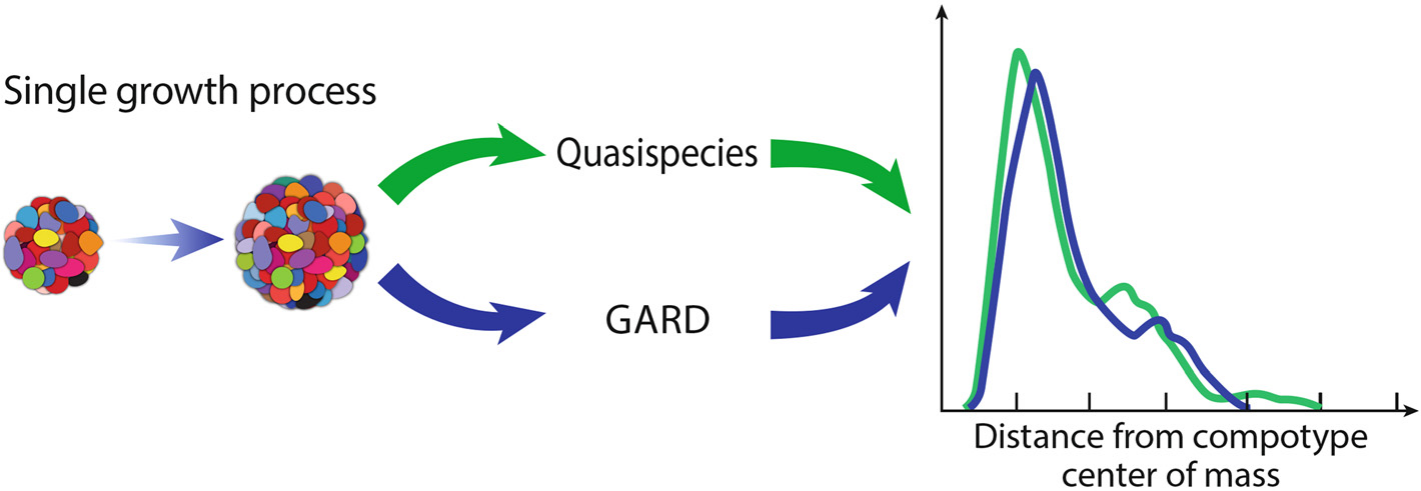
**Single growth process (SGP).** SGP is the common “generator” for both quasispecies and GARD. Using SGPs, the quasispecies and the GARD formalism are compared in this work. An SGP entails the growth via molecular accretion of a compositional assembly from size N_max_/2 to N_max_ (Methods).

The present manuscript attempts to show that compositional replicators, as described above, behave as quasispecies (Figure 1). As a model of compositional replication/reproduction, we employ the graded autocatalysis replication domain (GARD), a chemistry-based formalism that simulates network dynamics within amphiphile-containing compositional assemblies [36-38]. The GARD model quantitatively describes dynamics of out-of-equilibrium homeostatic growth, mediated by a network of mutual rate enhancement parameters with occasional assembly fission [36]. Molecules join assemblies, in a probabilistic fashion which is biased by a network of mutual rate enhancement parameters as dictated by assembly-composition, and occasional fission occurs such that an assembly is out-of-equilibrium [36]. GARD provides a detailed microscopic description of the walk in compositional space between the points representing molecular assemblies in a replication-like process. This is different from the quasispecies model, in which a microscopic view of replication is typically not provided.

GARD’s quasi-stationary states in compositional space are composomes, and their species-like clusters are compotypes [41]. The latter may serve as targets for selection [42], and exhibit ecology-like constant population dynamics [43].

As GARD assemblies store information in the form of non-random molecular compositions, and transfer this information to fission-generated progeny, they could be considered as alternative to the RNA world scenario for the origin of life [44-50]. To obtain a more complete picture of this proposed analogy, we asked whether GARD compositional assemblies may behave similarly to sequence-based quasispecies, despite the differences between the realms of sequence composition. We show that the cloud of compositional variants within a compotype obeys the quasispecies model and that it exhibits an error-catastrophe similar to the classical quasispecies.

## Results

### A compotype is qualitatively similar to a quasispecies

**Figure 2:**
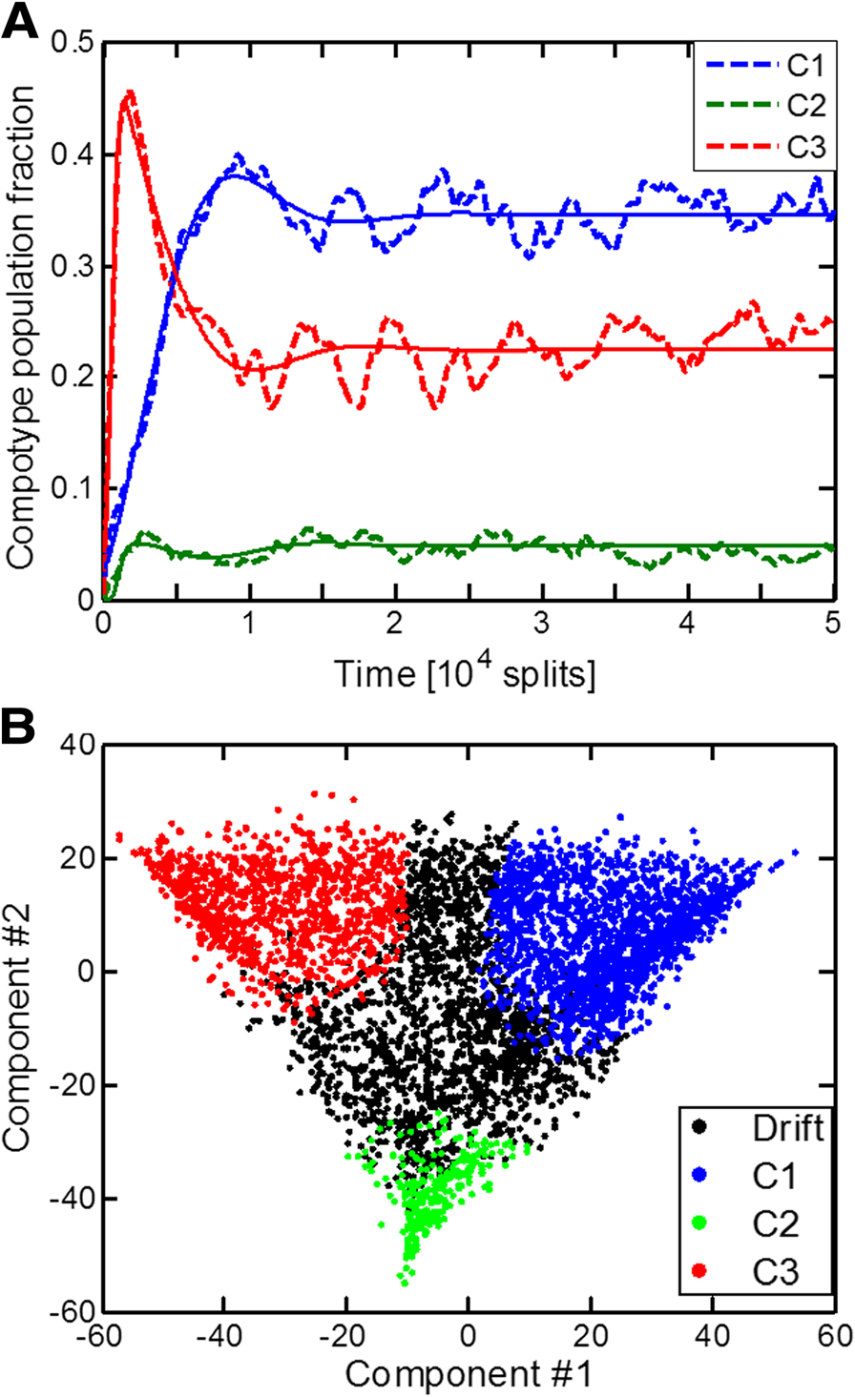
**GARD’s population dynamics results in a cloud in the compositional space. (A)** An example of a constant population dynamics (see Methods). This β exhibits 3 compotypes (N_C_ = 3). Solid lines are a fit to a multi-species logistic equation [43]. **(B)** Assemblies from the steady-state of this simulation (t = 4-5 × 10^4^) were subjected to a principle-component-analysis (PCA), and the top two axis are plotted. Each point represents an assembly and the color marks whether it belongs to a compotype or to drift.

**Figure 3:**
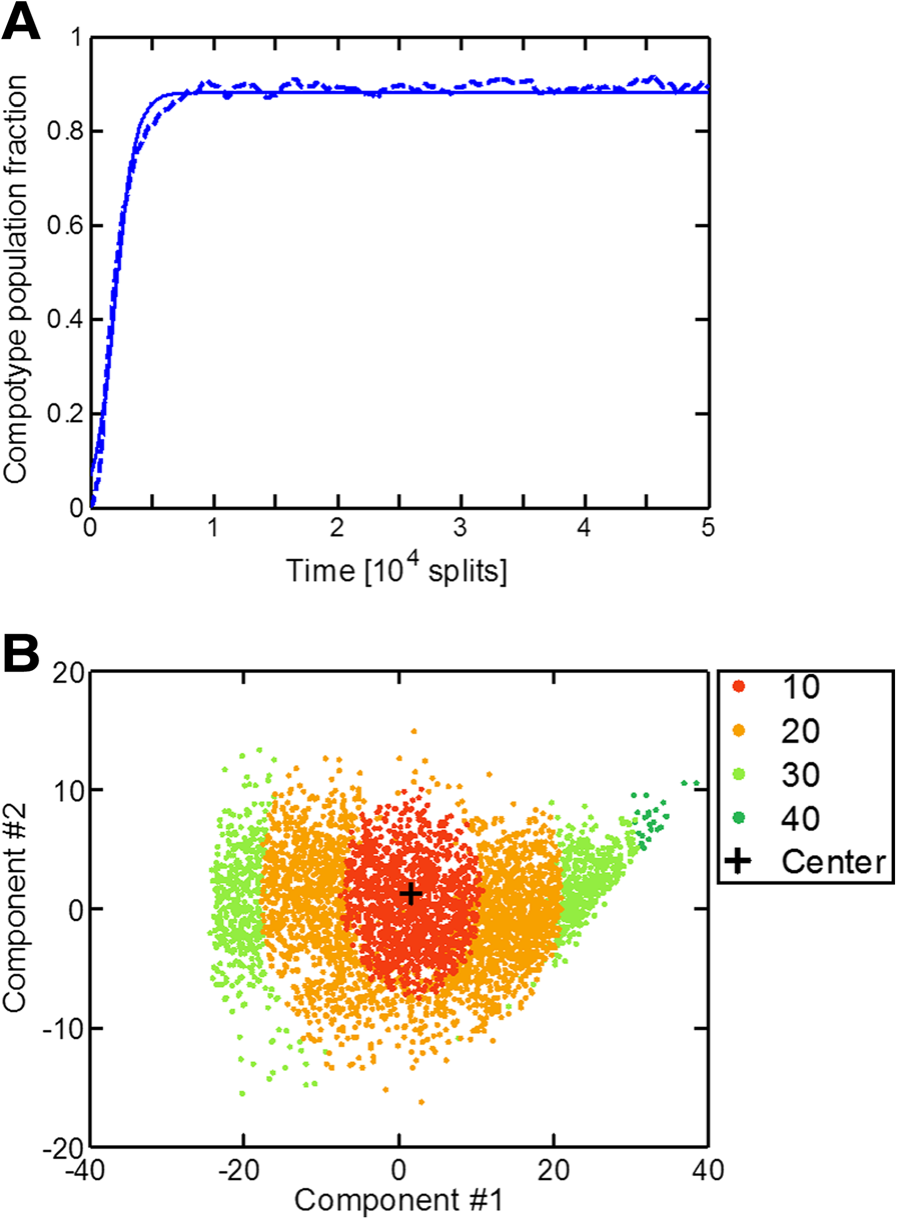
**The inner structure of GARD’s cloud. (A)** An example of a constant population dynamics, using a β which exhibits N_C_ = 1. **(B)** PCA of assemblies from this simulation’s steady-state. All the assemblies picked belong to the compotype and are colored according to their Euclidean distance from its center of mass. The black cross marks the location of the compotype center of mass after the PCA. All other details are as in Figure 2.

The GARD model depicts the dynamic behavior of a population of compositional assemblies. It portrays a “cloud” of compositional states, with dynamical interconversions (compositional mutations). Depending on the values of the rate enhancement parameters in β network (Equation 2), this may lead to cases with one or more compotypes (Figures 2 and 3). There are qualitative points of similarity between such compositional entities and quasispecies of sequence-based entities such as RNA molecules or viruses: both cases embody an ensemble of informational entities displaying a relatively high degree of mutual differences. Despite the similarities in the dynamics, the quasispecies and GARD equations are not identical. If each assembly-joining reaction is a Poisson process, GARD turns into a Markov chain (see Additional file 1: Supporting Data). The corresponding steady state of frequencies of different compositional assemblies is then linear, in contrast to the non-linear quasispecies equations. Those linear equations, however, require the complete set of all possible assemblies with all possible sizes (from 0 to N_max_), which is unattainable due to huge dimensionality of the system. It is the central empirical observation of this paper that the use of non-linear but rather simple quasi-species equations reproduces the statistics of GARD. While the complex reasons for this fact constitute a separate study, currently underway, here a numerical analysis is performed indicating that GARD is well-described by the quasispecies equations. The focus in the present study is on cases which exhibit only a single compotype each (N_C_ = 1), whereby compositional entities are disposed around a single center of mass (Figure 3), analogous to the master sequence in sequence-based quasispecies.

### Compotypes are quantitatively similar to sequence-based quasispecies

A central aim of the present paper is to provide evidence for quantitative similarities between the compositional assemblies and quasispecies in sequence space. For this, a total of almost 600 cases of GARD population steady states, each with N_C_ = 1, were analyzed. A simplifying principle in which groups of compositional assemblies with similar Euclidean distance to the compotype’s center of mass are lumped into “shells” was utilized (Figure 3). This is in analogy to certain quasispecies analysis, in which sequences with a similar Hamming distance towards the master sequence are lumped together [51]. This allowed deriving the compositional assemblies’ parameters for the quasispecies equation (Equation 1), and to compare the results from GARD’s simulations with those predicted from the quasispecies formalism. Due to the high dimensionality of the system (N_G_ = 100) the difference in volume between neighboring shells is enormous, which is why the results give the occupancy rather than the concentration of assemblies in each shell.

As a first step, a single growth process (SGP) is defined, which serves as a common “generator” for both the quasispecies and the GARD formalisms (Figure 1). An SGP entails the growth via molecular accretion of a compositional assembly from size N_max_/2 to N_max_ (Methods). For GARD simulations, this serves as an “atom” of the computational procedures that portray multiple growth and fission cycles in numerous assemblies in a reactor under constant population conditions [43]. For the quasispecies formalism, SGPs allow measuring the elements of Equation 1: the growth rates collected in the vector A and the transition probabilities collected in the matrix Q. Growth rates are obtained by a route analogous to the calculation of replication times in GARD populations ([43] and Methods). Transition probabilities from initial to final positions in compositional space are computed using SGPs (Methods). In other words, assemblies in the same distance shell are grouped together and the relevant properties (i.e. Q and A) of each shell are averaged over the assemblies contained in this shell. Fitness is defined as the rate of faithful replication (Q_ii_A_i_).

**Figure 4:**
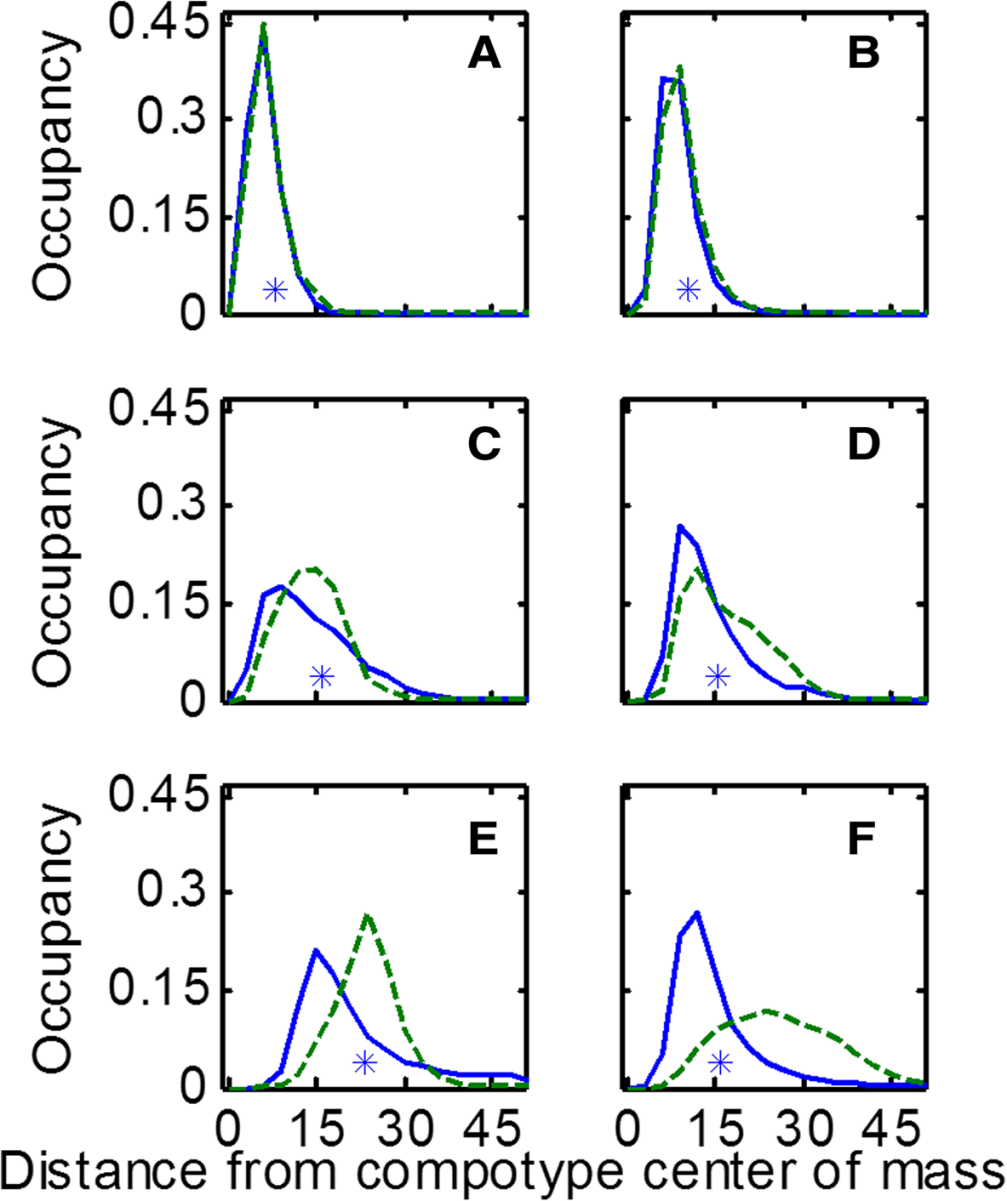
**Examples of steady state distance distributions of GARD vs. quasispecies.** Each panel shows an example of the distribution when measured from a particular GARD simulation (different β’s) and when calculated based on the quasispecies model. Blue solid line is GARD and green broken line is quasispecies. Asterisk marks the average distance of assemblies from the compotype center of mass in the simulation. Fitness landscapes of these β’s are given in the Additional file 1: Supporting Data. Each shell thickness (i.e. bin width) = 3. The estimated effective volume of the compositional space in each panel is proportional to: 3 × 10^125^, 1 × 10^138^, 5 × 10^147^, 4 × 10^155^, 1 × 10^159^ and 6 × 10^170^, respectively. Lognormal seeds used for generating these β’s are: 49, 8, 1, 6, 37 and 21, respectively for panels **A-F**.

**Figure 5:**
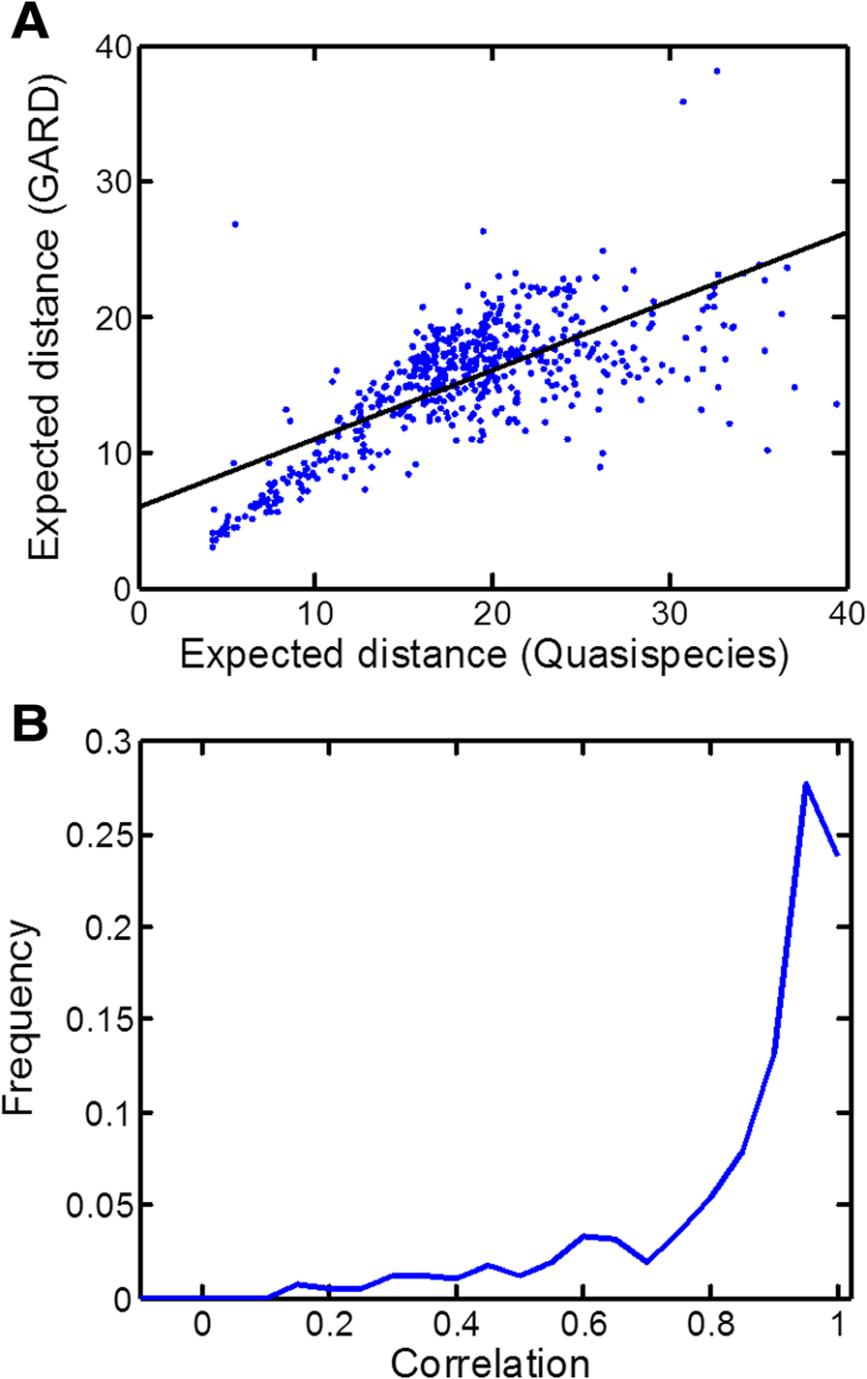
**GARD is similar to quasispecies.** A widespread comparison of GARD vs. quasispecies, based on 571 simulations. **(A)** The expected distance (=∫p(r)dr) of each of distribution of GARD vs. quasispecies. Black solid line is linear fit: y = 0.507*x + 5.96, R = 0.717. **(B)** Each pair of distributions is subjected to a Pearson-correlation (see examples in Additional file 1: Supporting Data), and this panel shows the histogram of these correlation coefficients. 75% of cases show high correlation (>0.8), with high mean correlation (0.85 ± 0.18).

Once A and Q are populated, it is straightforward to employ the quasispecies formalism in order to compute the steady state distribution of fractional occupancy of assemblies within the different distance shells. In parallel, the same distribution is computed based on the full-fledged GARD model, essentially a long series of single growth process followed by fission events [43]. Rewardingly, the distributions obtained by both methods portrayed a high degree of similarity (Figures 4 and 5). Such results support the notion of inherent resemblance between the presently analyzed compositional entities and the classical constituents of quasispecies, namely sequence-based entities.

Importantly, such a good agreement between the distributions is obtained only when A and Q are measured with respect to the compotype (which is an attractor in the compositional space, see Additional file 1: Supporting Data), whereas comparing the distributions with respect to a random assembly or even the eigenvector of β results in a meager agreement (p-values 6.38 × 10^−7^ and 4.75 × 10^−8^, respectively. See Additional file 1: Supporting Data).

### Similar time dependent dynamics for GARD and for quasispecies

**Figure 6:**
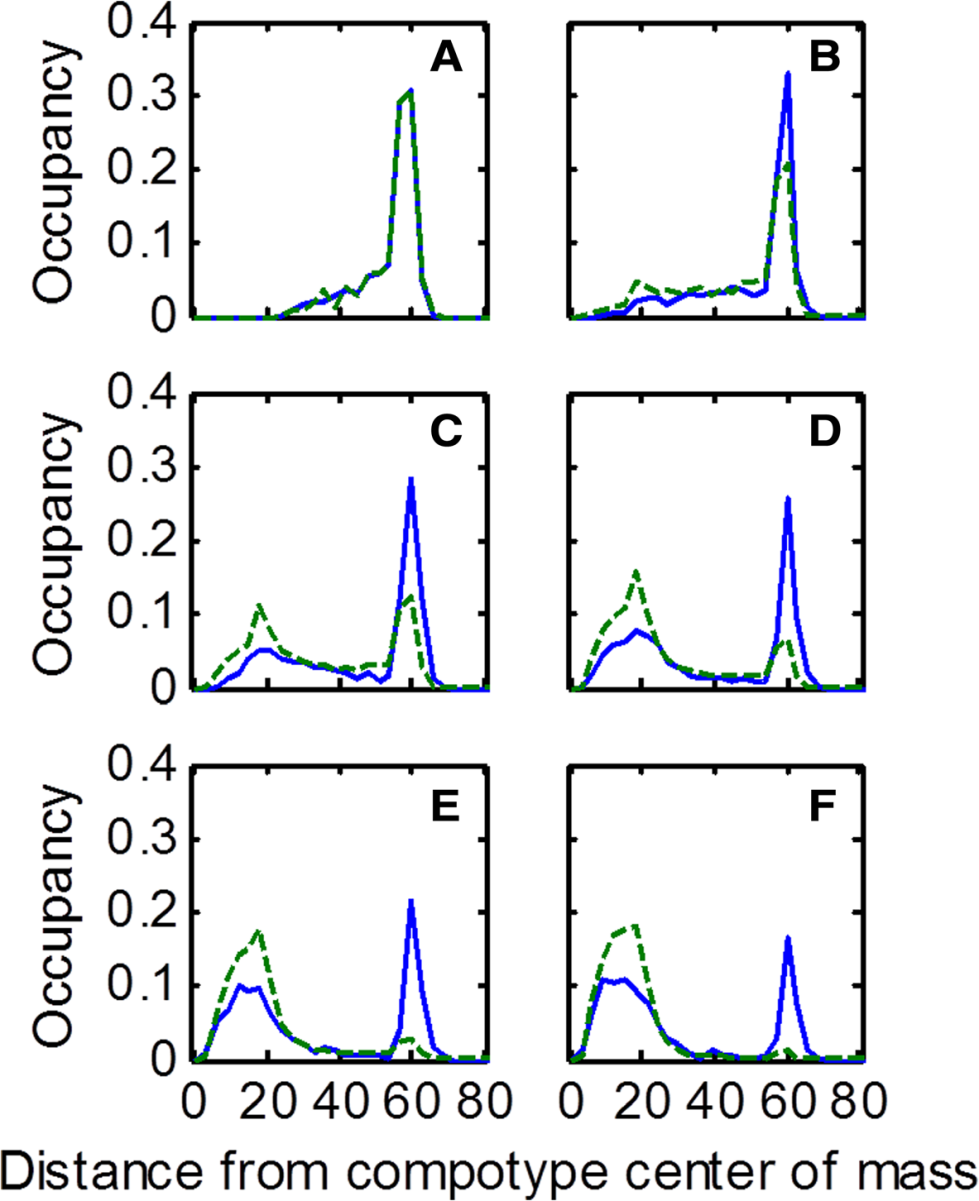
**An example of the dynamics towards steady-state.** Each panel shows GARD’s and quasispecies’ distance distributions at different times. Both GARD’s and quasispecies’ time dependent dynamics show the same behavior, where the steady state peak at distance = 15 grows at the expanse of the peak at distance = 60. GARD’s time dependent dynamics were sampled at fixed time intervals from t = 0 **(panel A)** to a time close to steady state **(panel F)**. In parallel, the same was repeated for quasispecies. Lognormal seed used for generating this β is 1. Further details are given in the Additional file 1: Supporting Data.

**Figure 7:**
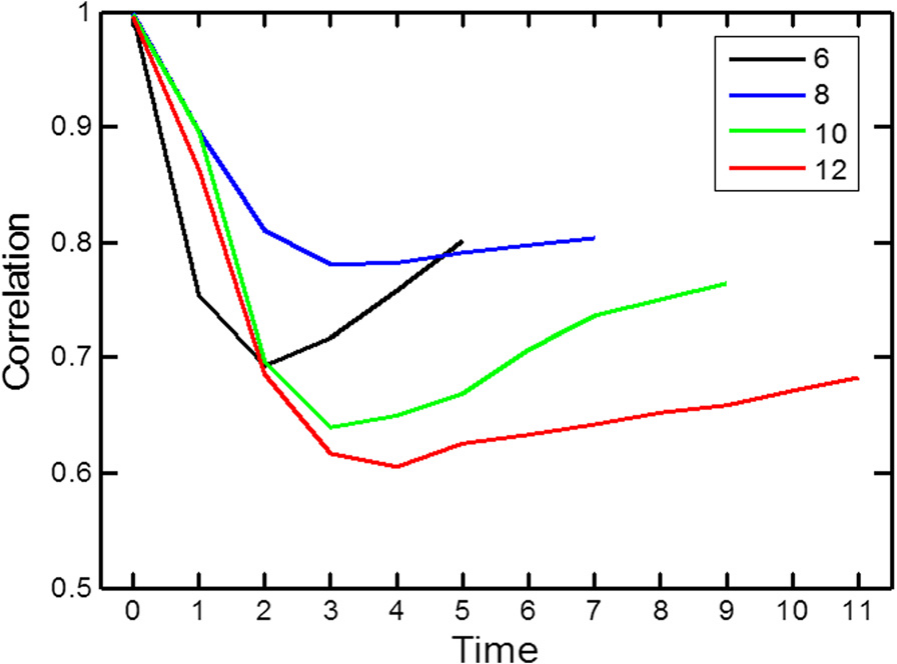
**GARD’s time dependent dynamics are similar to quasispecies’.** This figure shows the Pearson correlation coefficient for the time dependent dynamics, for groups of simulations that reached steady state after 6, 8, 10 and 12 time points. Further details are given in the Additional file 1: Supporting Data.

It is asked whether the similarity of dynamic behavior transcends the steady state distributions. For that, the time dependent evolution of the fractional occupancy distribution between the GARD and the quasispecies equation were compared. In both cases the computation started from the same initial conditions, and the system was allowed to propagate towards steady state. The time development as predicted from the GARD equations showed appreciable similarity to that predicted by the quasispecies equation (Figures 6 and 7). This lends further support to the mutual resemblance of the two models.

### Compositional error threshold

**Figure 8:**
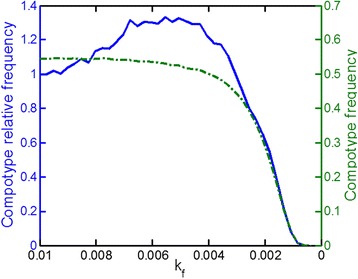
**Compotype average frequency and relative frequency for increased error-rates.** For each k_f_ value, all simulations were run in a single-lineage mode for 2,000 generations and the frequencies were recorded. Relative frequency is measured against the frequency with the typical value of k_f_ = 0.01. This figure shows the average across all simulations.

It is asked whether compositional entities, as described by GARD, may manifest an error threshold, in resemblance to sequence-based entities in the quasispecies model. For this a quantitative analog of the global mutation rate was sought. A change in such a parameter should show a graded diversification of the compositional vectors away from the compotype’s center of mass, eventually leading to a dismantle of the compotype structure. It is discovered that one of the basic rate constants of the GARD model, k_f_, the basal molecular joining rate (Equation 2), is a suitable proxy. Decreasing k_f_ results in an overall diminution of assembly growth, leading to a predominance of the backward (assembly-exit) reactions governed by k_b_. This results in an enhanced probability of amphiphile mis-incorporation, and hence increased compositional mutations. Indeed, as k_f_ diminishes by a factor of 10, the assembly population typically strays away and the assembly fraction residing within the compotype boundaries goes to 0 (Figure 8).

**Figure 9:**
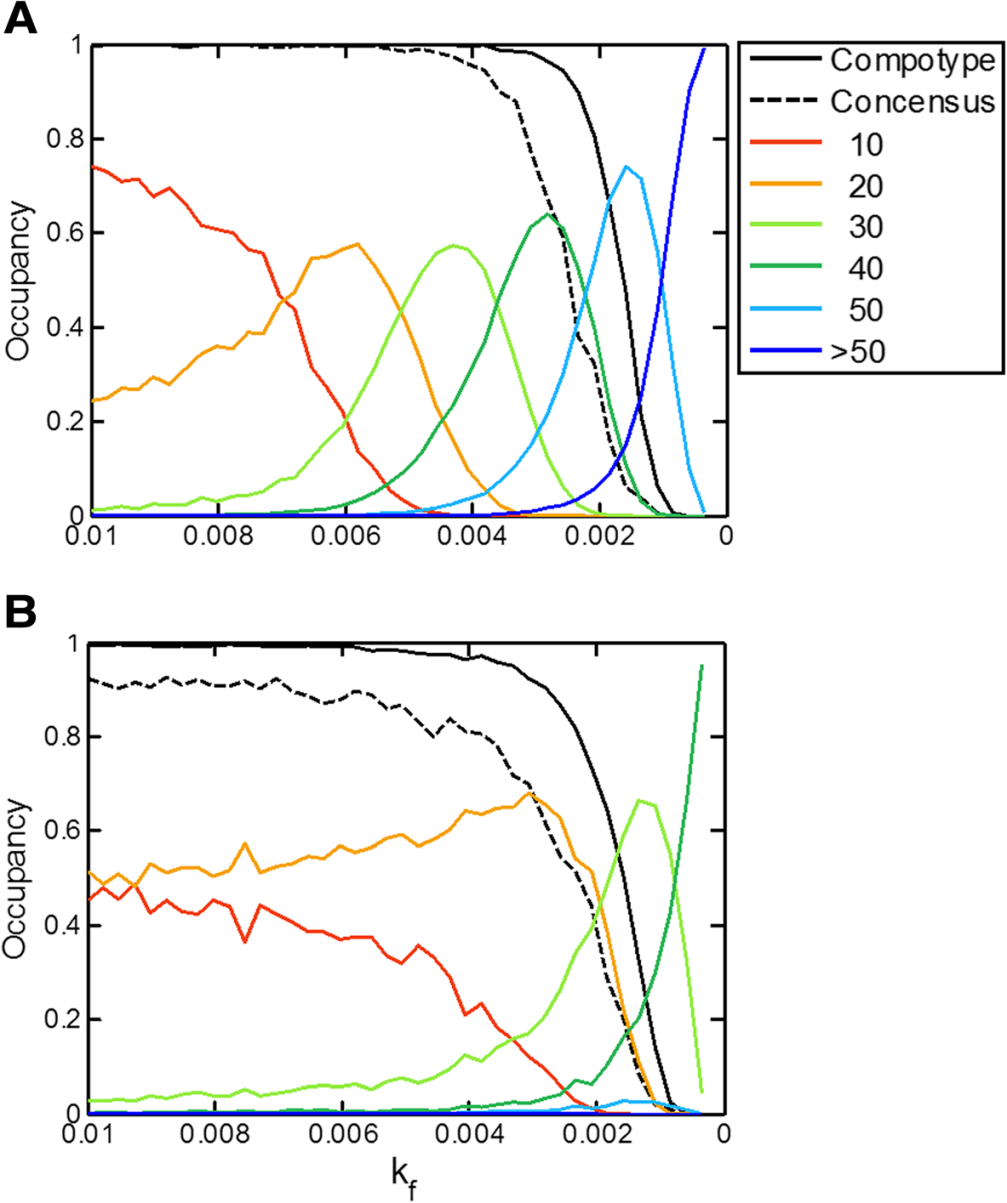
**An example of GARD’s error catastrophe.** Compotype frequency (black) and occupancy at increasing distance shells (colors) for decreasing k_f_ values. Lognormal seeds used for generating these β’s are 49 for panel **A** and 8 for **B**. Each shell thickness = 5. Similarly to a consensus-sequence [7,51], the consensus-composition is a vector, where each molecular-type i is assigned with the most probable n_i_ value within the compotype member assemblies.

When k_f_ was gradually diminished, a behavior reminiscent to that of classical error catastrophe in sequence-based quasispecies [51] (Figure 9). With decreasing k_f_, the occupancy of increasingly distant compositional shells was enhanced and then diminished. Beyond a specific range of k_f_ values there was a relatively sharp decline of the compotype occupancy, similar to the sharp decline of the consensus sequence in sequence-based quasispecies. The specific shape of this response to decreasing kf depends on the fitness landscape in each simulation (which is an emergent property of β).

## Discussion

The present work aimed at showing that compositional replicators may behave as quasispecies. For this, the graded autocatalysis replication domain (GARD) model, which simulates the kinetics of amphiphile-containing compositional assemblies, was employed. GARD was originally developed in an attempt to bridge between the “genetic-first” and the “metabolism-first” scenarios for the origin of life [37]. The genetic- (or replicator-) first scenario, also known as “information first” scenario, assumes that a molecule identical or very similar to present day RNA played the role of the self-perpetuating biopolymer [44,46,52,53]. The “free-floating” or surface-adsorbed mixture of such molecules is assumed to have later evolved both a metabolic network and an encompassing container. The metabolism-first scenario suggests that the very first life precursors are likely to have been relatively elaborate molecular networks of simple molecules [38,45,48,54,55]. The GARD model is basically about small molecules, resembling those typically considered as metabolites, which when accreting into molecular assemblies portray a dynamic behavior resembling that of replicators. When doing so, GARD assemblies utilize an unorthodox form of information transfer, namely, the propagation of compositional information.

An error threshold is a hallmark of quasispecies dynamics. In the case of sequence-based quasispecies, one of the parameter that influences this threshold is polymer length, whereby longer polymers show higher error threshold susceptibility [4,56]. In our analyses a more facile approach to error threshold is observed when diminishing k_f_, the basal rate of monomer joining into a molecular assembly. It may be asked whether, as a parallelism, GARD error threshold could be related to an assembly size parameter. Previously, it was shown that for a given N_G_, diminishing the maximal assembly size (N_max_) results in higher compotype diversity [42]. This may be interpreted as occurring via compositional mutations as described [57]. Thus, an enhanced mutability via reduced N_max_ is suggested as a good candidate proxy to increasing polymer length in the context of an error catastrophe. Future detailed analyses could provide support to this notion.

## Conclusions

In conclusion, molecular assemblies that hold compositional information rather than sequence-based information are shown here to comply with a quasispecies description. Because the transmission of compositional information has been proposed to be important in early evolution, these results further underline the importance of the quasispecies model in studying prebiotic evolution. Further, because present-day cells are, in many ways, compositional entities, such results may also have implications to the understanding of populations of present-day cells.

## Methods

### The Eigen-Schuster quasispecies equations

The quasispecies formalism describes a population of self-replicating genotypes (Equation 1) [2-4]. Due to replication errors, a genotype produces not only offspring of its own kind, but might also produce offspring of other genotypes. This is represented by the transition matrix (Q) which denotes the probability at which a certain genotype will produce an offspring of another genotype. Thus, the growth of a particular genotype is governed not only by its own replication rate, but also by the replication rate of the other genotypes. The quasispecies equation is written as:

1 $$ \frac{d{x}_i}{dt}={\displaystyle \sum_j{A}_j{\mathrm{Q}}_{ij}{x}_j}-\tilde{E}(t){x}_i $$

Where for a genotype i, x_i_ is its time dependent concentration, A_i_ is its replication rate (as it reflects its fitness [3]) and Q_ij_ is the probability of genotype j mutating into i (with Q_ii_ being the probability of self-replication). Ĕ(t) = ∑x_i_A_i_ is termed “average excess rate” and serves to keep the total population size constant (∑x_i_ = 1 at all time points). A steady-state solution to this equation is obtained as the eigenvector with largest eigenvalue of the matrix W = {Q⋅diag(A)} (where (diag(A) is a matrix whose values along the diagonal are the values of the A vector, and zero otherwise), in accordance to Perron-Frobenius theorem [3,58]. This eigenvector holds the occurrences fractional occupancy of phenotypes at steady-state, which are the quasispecies.

### The GARD model

**Figure 10:**
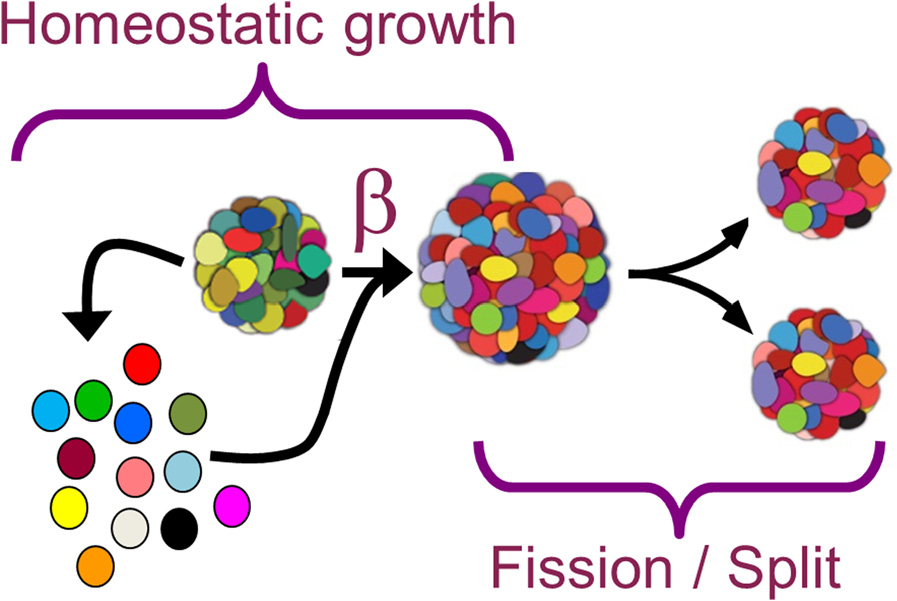
**A general scheme of the GARD model.** The environment contains a large, buffered, repertoire of different amphiphiles. An assembly forms and grows by the accretion of amphiphiles, which is dictated by network of rate-enhancement parameters (β, which represents the environmental chemistry) and the assembly’s composition. Once it has reached a predefined maximal size, a binary fission occurs and the growth cycle can begins again.

GARD is a kinetic model which describes the growth and fission of a molecular assembly (Figure 10 is a scheme of the model), typically assumed to consist of a repertoire of N_G_ amphiphilic molecule types (environmental repertoire) [36]. Molecules from a buffered environment form and join an assembly and molecules within it can leave. Once the number of molecules in an assembly reaches a pre-defined maximal size (N_max_), a random fission action is applied to produce two progenies of same size (N_max_/2) which can grow again and again in growth-fission cycles. The dynamics are described by a set of ordinary differential equations:

2 $$ \frac{d{n}_i}{dt}=\left({k}_f{\rho}_iN-{k}_b{n}_i\right)\ \left(1+{\displaystyle \sum_{j=1}^{N_G}{\beta}_{ij}}\frac{n_j}{N}\right) $$

Where ni is the current count of molecule type i in an assembly (i = 1..N_G_), k_f_ and k_b_ are the basal forward and backward rate constants (assembly joining and leaving, respectively), ρ_i_ is the buffered environmental concentration and N is current assembly size (N = ∑n_i_). β_ij_ is the rate-enhancement exerted by an assembly molecule of type j on incoming or outgoing molecule of type i. β can be represented as N_G_ × N_G_ matrix or as network with N_G_ nodes and N_G_^2^ edges [42], where different β instances represent different environmental chemistries. Typically, GARD is run in a single-lineage mode, where at each split event only one progeny (picked at random) is followed and the other one is discarded [36].

A composome is defined as a set of subsequently faithfully replicating assemblies (a term originally derived from compositional genome), where a faithfully replicating assembly is defined as an assembly which is highly similar to its predecessor and successor, when GARD is run in single-lineage mode [36]. Similar composomes are grouped into a compotype, using K-means clustering algorithm [41]. A compotype is represented by a compositional vector constituting the center of mass of all its member assemblies.

When β is represented in the matrix form, it is a positive matrix, as each of its β_ij_ values are sampled from a lognormal distribution [59]. According to the Perron-Frobenius theorem, such a matrix has a unique largest real eigenvalue with a corresponding all positive real eigenvector [58]. The eigenvector was treated as a compositional assembly and marked V_β_.

### Single-growth-process

A SGP is complete single cycle, leading from an assembly at size N_max_ to a following assembly at N_max_ (Figures 1 and 10). It is performed as follows: a parent assembly is picked at size N_max_; the parent than undergoes fission to produce a progeny at size N_max_/2 (see comment below); this progeny is then grown to size N_max_ according to the GARD equations (Equation 2) and the SGP is complete. A SGP tracks only one of the progeny, and tracking the other progeny is considered an additional SGP.

### GARD simulations

The GARD10 MATLAB code was used for all simulations [42]. Different simulations were run using identical parameters but with different β networks, generated by the MATLAB pseudorandom number generator with different random seeds. When addressing GARD’s population dynamics (population-GARD), dataset was obtained from [43]. In population-GARD, each simulation represents a chemostat which is initially seeded with 1,000 random compositions. Assemblies are allowed to simultaneously grow based on their idiosyncratic kinetic parameters, while the total size of the population is maintained constant, based on a Moran process [60]. This was done for a total of 50,000 SGPs and the sampling of GARD distance distribution was done by collecting the states of the chemostat along the population steady state (t = 4.9-5.0 × 10^4^ with time intervals of 0.1 × 10^4^. See for example Figure 1 in [43]). Depending on the values of the rate enhancement parameters in β, different simulations exhibit one or more compotypes [42]. The focus in the present study is on 572 cases which portray only a single compotype each (N_C_ = 1).

### Sampling the compositional space and constructing Q and A

The large size of the compositional space, particularly given the values used in this work, N_G_ = 100 and N_max_ = 100, makes direct calculation of Q matrix computationally impossible. Therefore, the N_G_-dimensional molecular space was divided into shells of constant thickness, centered on the compotype center of mass, and assemblies were grouped according to their Euclidean distance from the center of mass (Equation 3). This is in contrast to a previous study [61], where the Q and A vector where directly calculated by using substantially different N_G_ and N_max_ values than those typically employed in GARD, which enabled direct enumeration of the small number of possible compositions.

The Euclidean distance between two assemblies is calculated as:

3 $$ r\left({V}^1,{V}^2\right)=\sqrt{{\displaystyle \sum_{i=1}^{N_G}{\left({n}_i^1-{n}_i^2\right)}^2}} $$

Where n_i_^1^ is the count of the i’th molecular type in assembly V^1^. The maximum possible distance between any two assemblies is N_max_√2.

Assemblies in the same distance shell were grouped together and the relevant properties (i.e. Q and A) of each shell were averaged over the assemblies contained in this shell. Q_ij_ is then the average probability that a parent at distance shell j will gave rise to a progeny at shell i after a single SGP, and A_i_ is the average growth rate of progenies at shell i.

For each simulation, the compositional space was sampled by performing 600,000 SGPs based on 30,000 parent assemblies, as detailed:

10,000 parent assemblies were generated at random, each by randomly picking a molecular type and adding a random count of this type until the assembly size reaches N_max_. Another 10,000 parents were generated by conducting 10,000 random walk step pairs starting from the compotype center of mass, where in each step a molecule is randomly removed from the assembly and a random one is added to it. Another 10,000 parents were generated by random walk starting from the V_β_. Then, for each parent, 20 SGP were performed, each beginning with the parent assembly. Examples of Q and A are given in additional file 1: Supporting Data.

## Additional file

Additional file 1:
**Supporting Data.** Additional text, mathematical analysis and figures supporting the results of this article.

## Abbreviations

GARD: Graded autocatalysis replication domain
SGP: Single growth process
