# Supplementary material for: Quasispecies in population of compositional assemblies
Source: BMC Evol Biol. 2014 Dec 30;14:265. doi: 10.1186/s12862-014-0265-1 (PMC4357159; doi:10.1186/s12862-014-0265-1)
Supplement: Additional file 1: — Supporting Data. Additional text, mathematical analysis and figures supporting the results of this article. [file 12862_2014_265_MOESM1_ESM.docx]

**Quasispecies in population of compositional assemblies – additional file 1: Supporting Data**

Renan Gross, Itzhak Fouxon, Doron Lancet and Omer Markovitch*

* Corresponding author: omermar@gmail.com

1. **A mathematical derivation of GARD’s transition matrix and steady state**

GARD provides a detailed microscopic description of the walk in compositional space between the points representing molecular assemblies in a replication-like process. This is different from the quasispecies model, in which a microscopic view of replication is typically not provided. In order to offer a basis to analytically compare the two models, GARD (Equation 2 in the main text) will now be considered on a coarse-grained time scale (∆t) which is much larger than that of a typical single molecule joining or leaving. This allows describing the 2N_G_ possible reactions (N_G_ joining and N_G_ leaving) as independent Poisson processes. The probability of joining and leaving is then $\Gamma_{i}^{+}\Delta t$ and $\Gamma_{i}^{-}\Delta t$, respectively, where:

$$\begin{matrix} \Gamma_{i}^{+}\equiv\Gamma_{i}^{+}[(n_{1},n_{2},...,n_{N_{G}})]=k_{f}\rho_{i}N\text{ }\left(1+\sum_{j=1}^{N_{G}} \beta_{ij}\frac{n_{j}}{N}\right) \\ \Gamma_{i}^{-}\equiv\Gamma_{i}^{-}[(n_{1},n_{2},...,n_{N_{G}})]=k_{b}n_{i}\text{ }\left(1+\sum_{j=1}^{N_{G}} \beta_{ij}\frac{n_{j}}{N}\right) \end{matrix}$$

Equation S1

GARD’s equation (Equation 2 in the main text) is: $\Gamma_{i}^{+}-\Gamma_{i}^{-}$.

The change in the frequency of an assembly $x_{\left(n_{1},n_{2},...,n_{N_{G}}\right)\text{ }}$which has a composition $(n_{1},n_{2},..,n_{N_{G}})$ can now be written as:

$$\begin{matrix} \frac{dx_{\left(n_{1},n_{2},...,n_{N_{G}}\right)\text{ }}}{dt}=\sum_{i=1}^{N_{G}} \Gamma_{i}^{+}[(n_{1},n_{2},..,n_{i-1},..,n_{N_{G}})]x_{\left(n_{1},n_{2,}..,n_{i}-1,..,n_{N_{G}}\right)} \\ +\sum_{i=1}^{N_{G}} \Gamma_{i}^{-}[(n_{1},n_{2},..,n_{i+1},..,n_{N_{G}})]x_{\left(n_{1},n_{2,}..,n_{i}+1,..,n_{N_{G}}\right)}-\frac{x_{\left(n_{1},n_{2},...,n_{N_{G}}\right)\text{ }}}{\tau[(n_{1},n_{2},...,n_{N_{G}})]\text{ }} \end{matrix}$$

Equation S2

where the first and the second terms on the right hand side describe the contribution of assemblies with compositions $\left(n_{1},n_{2,}..,n_{i}-1,..,n_{N_{G}}\right)$ and $\left(n_{1},n_{2,}..,n_{i}+1,..,n_{N_{G}}\right)$ that respectively have one less and one more of the i-th molecule type than $x_{\left(n_{1},n_{2,},..,n_{N_{G}}\right)}$. Thus, the first term on the right-hand-side describes the increase of the $(n_{1},n_{2},..,n_{N_{G}})$’s frequency due to the joining a molecule of type i to assemblies with lower molecular counts, the second term describes increase due to a molecule of type i leaving assemblies with higher molecular counts and the last term describes decrease due to the events where the assemblies with molecular compositions $(n_{1},n_{2},..,n_{N_{G}})$ which are changing due to joining or leaving of the molecules where the decay time *τ* obeys:

$$\tau^{-1}[(n_{1},n_{2},...,n_{N_{G}})]=\sum_{i=1}^{N_{G}} (\Gamma_{i}^{+}[(n_{1},n_{2},...,n_{N_{G}})]+\Gamma_{i}^{-}[(n_{1},n_{2},...,n_{N_{G}})])$$

Equation S3

Since GARD assumes assemblies of size N_max_ split instantaneously, then for these assemblies the frequency (fraction of time the assembly spends in that state) is zero. With this, Equation S2 now describes the time-dependent frequencies of all assemblies except for those that have size N_max_/2. For the latter assemblies the equation is:

$$\begin{matrix} \frac{dx_{\left(n_{1},n_{2},...,n_{N_{G}}\right)\text{ }}}{dt}=\sum_{i=1}^{N_{G}} \Gamma_{i}^{+}[(n_{1},n_{2},..,n_{i-1},..,n_{N_{G}})]x_{\left(n_{1},n_{2,}..,n_{i}-1,..,n_{N_{G}}\right)} \\ +\sum_{i=1}^{N_{G}} \Gamma_{i}^{-}[(n_{1},n_{2},..,n_{i+1},..,n_{N_{G}})]x_{\left(n_{1},n_{2,}..,n_{i}+1,..,n_{N_{G}}\right)}-\frac{x_{\left(n_{1},n_{2},...,n_{N_{G}}\right)\text{ }}}{\tau[(n_{1},n_{2},...,n_{N_{G}})]\text{ }} \\ +\sum_{\begin{matrix} i=1 \\ \Sigma n'_{i}=N_{\max} \end{matrix}}^{N_{G}} \Gamma_{i}^{+}[(n'_{1},n'_{2},..,n'_{i-1},..,n'_{N_{G}})]x_{\left(n'_{1},n'_{2,}..,n'_{i}-1,..,n'_{N_{G}}\right)}P_{f}\left(n'\to n\right) \end{matrix}$$

Equation S4

where the last term describes the creation of assemblies of size N_max_/2 by the random fission of assemblies of size N_max_. Here $P_{f}(n'\to n)$ is the corresponding transition probability that obeys the multivariate hypergeometric distribution [Y. M. Bishop, S. E. Fienberg, and P. W. Holland, *Discrete Multivariate Analysis: Theory and Practice*, Springer (2007)].

The equation on x_p_, where p stands for the composition $(n_{1},n_{2},...,n_{N_{G}})$ can be written in the vector form:

$$\frac{dx_{p\text{ }}}{dt}=\sum_{q} U_{pq}x_{q}$$

Equation S5

Thus, GARD is now turned into a Markov chain with a transition matrix U. The steady state is the eigenvector of *U* with an eigenvalue of 0. This is because at steady state the left-hand-side of Equation S5 equals zero. However, due to overwhelming number of compositions for our typical N_G_=100 and N_max_=100 (about exp(138)), it is impossible to compute analytically the steady state values. For this reason, the present manuscript compares results from reduced-dimensionality GARD-based simulations to those computed for the quasispecies model.

1. **A compotype is an attractor in the compositional space**

shows the transition matrix, Q, when averaged over the entire set of 571 simulations performed. The most striking feature is that for all but the smallest distances, replication occurs towards the compotype’s center of mass (i.e., for a certain from-distance, the highest transition probability is of a to-distance<from-distance). For Euclidean distances>40, replication always occurs towards the center of mass, while for intermediate distances between 20 and 40 replication can occur towards and away from it, and for distance<15 replication is usually away from it. In other words, the progeny of any parent assembly located far (distance>40) from the compotype’s center of mass will always grow to be closer to it than the parent, while a parent located very close (distance<15) to it will grow to be slightly further away. Thus, a compotype is an attractor in the N_G_-dimensional compositional space.


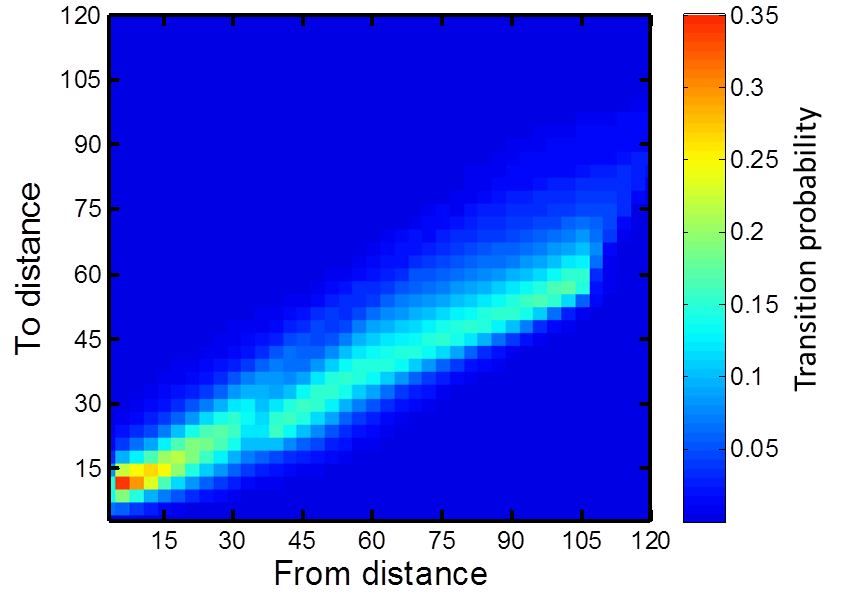


Figure S1: A compotype is an attractor in the N_G_-dimensional compositional space. This figure shows the average Q. For specific examples see .

1. **Comparing the steady-state distance distributions with respect to additional assemblies**

While the distance distributions measured from GARD and calculated using the Eigen-Schuster equations agree nicely, there is a need to verify if such an agreement can occur with other compositional assemblies or only for compotypes.

For a set of 41 simulations, the entire process of populating Q and A and calculating the steady-state distribution from the Eigen-Schuster equations was repeated, in an identical procedure to as before. This was done for two cases: one where the point of reference was a random assembly instead of the compotype center of mass, and the other where the point of reference was V_β_ (see Methods). In parallel, GARD’s distribution was re-measured with respect to these two assemblies. This gave rise to two datasets, each consisting of 41 pairs of steady-state distance distributions.

The mean Pearson correlation coefficient for the cases using a random assembly is 0.351±0.407 and for V_β_ is 0.538±0.341. With respect to the compotype, the mean value of the coefficient is substantially higher, 0.884±132. Additionally, a 2-samples Kolmogorov-Smirnov test rejected the null hypothesis that the new sets are similar to the compotype-set, with p-values of 6.38×10^-7^ and 4.75×10^-8^, respectively for the random-assembly and V_β_.

1. **Examples of Q and A**

and respectively show Q and A (Equation 1 in the main text) for the 6 examples given in the main text (Figure 4).


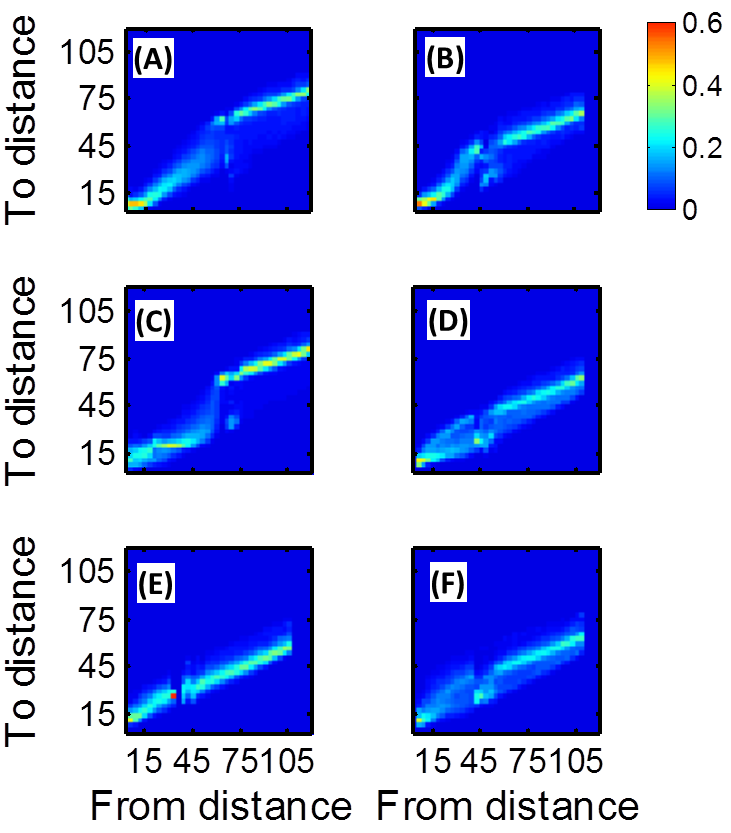


Figure S2: Examples of transition matrix (Q). Color represents the transition probability. Each shell thickness (i.e. bin width) = 3.


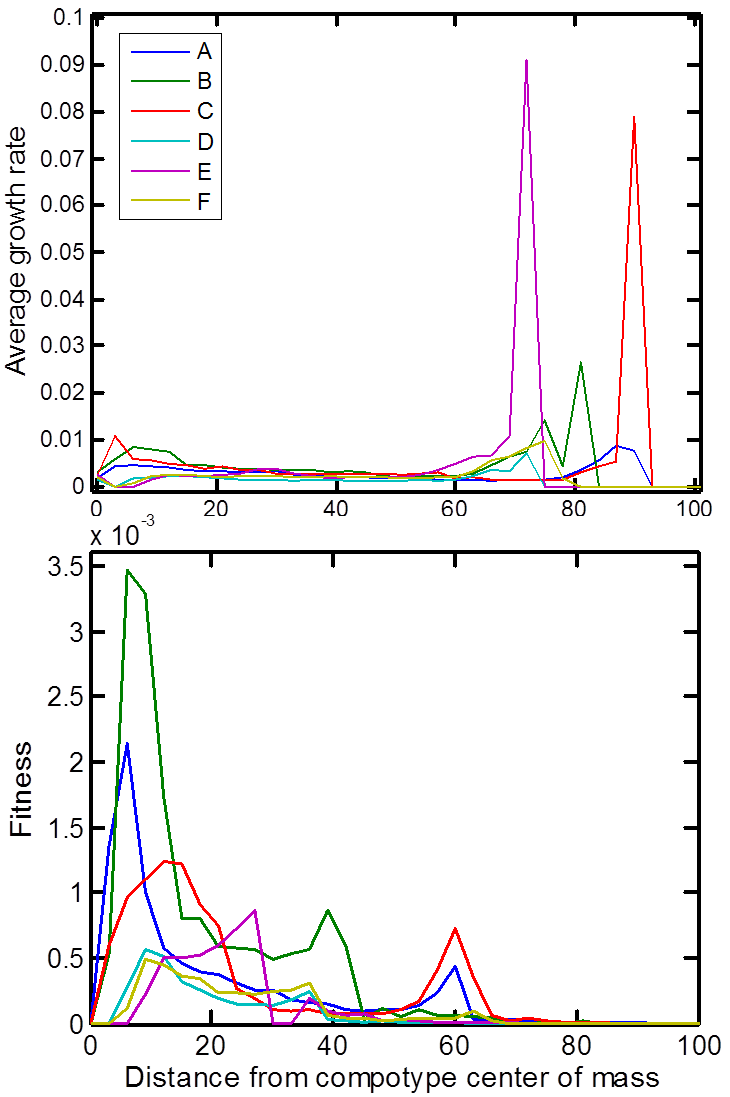


Figure S3: Examples of growth rate (A) and fitness. Fitness is defined as Q_ii_A_i_. Each shell thickness = 3.

1. **Examples Pearson correlation calculation**

For each β studied, two steady state distributions were generated – GARD’s and quasispecies’. For each pair of distributions, the Y axis represents the GARD data and the X axis represents the quasispecies data (). The Pearson correlation is than the value of the correlation coefficient of a linear fit (using MATLAB’s *corr* function).


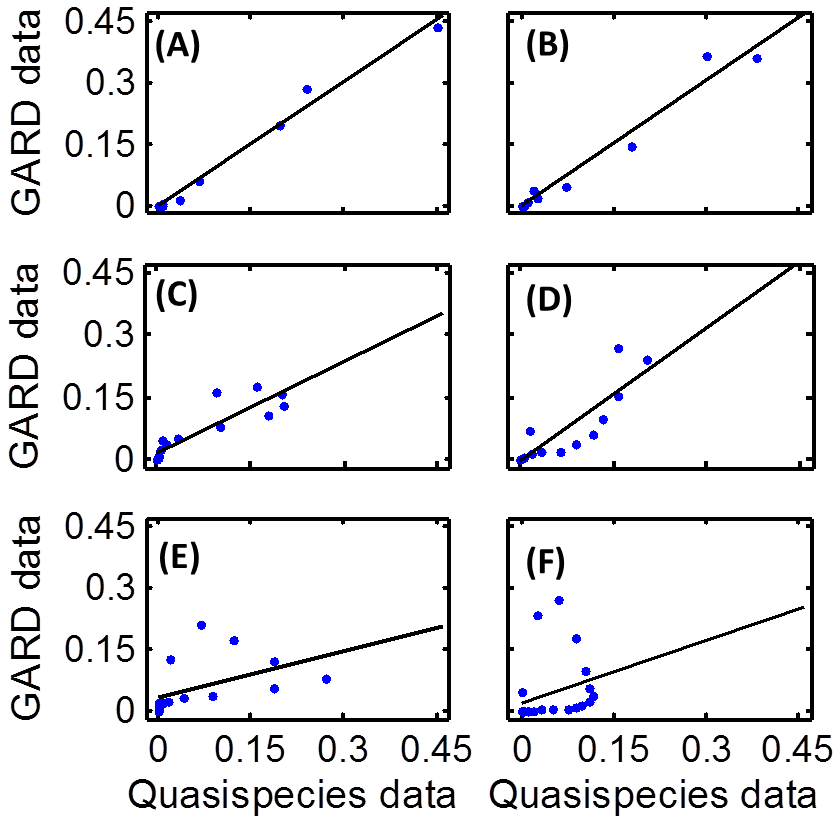


**Figure S4: Examples of Pearson correlations between GARD’s and quasispecies’ distance distributions**. These examples match the 6 examples given in the main text (Figure 4). Solid black line represents linear fit.

1. **How the time dependent dynamics were analyzed**

Starting at t=0, at each time point the distance distribution was calculated from GARD’s population-dynamics data [Markovitch and Lancet (2014)]. Then, the root-mean-square-deviation (RMSD) between each pair of distributions from t and t+1 was calculated. At each time point t, the variance in the RMSD was calculated as the variance of all the time points until the time t. Finaly, the steady state time was evaluated as the first time point, after the maximum, were the variance reached half of its maximal value. In parallel, this was repeated for quasispecies time dependent dynamics (Equation 1). This gave rise to two time intervals were GARD and quasispecies respectively reached steady state.

These time intervals were than divided such that the GARD and quasispecies intervals will have the same number of points, and at each of these points the Pearson correlation coefficient was calculated.
